# Understanding the Impact of Livelihood Diversification on Food Security Among Smallholder Farmers in West Gojjam Zone, Amhara Region, Ethiopia

**DOI:** 10.1155/tswj/6095651

**Published:** 2025-09-12

**Authors:** Silabat Enyew Zewudie

**Affiliations:** Department of Economics, Debre Markos University Bure Campus, Debre Markos, Amhara Region, Ethiopia

**Keywords:** food security, livelihood diversification, logistic regression, propensity score matching, West Gojjam Zone

## Abstract

**Background:** Livelihood diversification is widely recognized as a vital strategy for improving food security among smallholder rural households. However, achieving meaningful diversification remains a challenge due to various socioeconomic and institutional constraints. This study investigates the impact of livelihood diversification on food security in the West Gojjam Zone of Ethiopia.

**Methods:** A cross-sectional research design was employed using primary data collected from 390 randomly selected smallholder farmers through a multistage sampling technique. Binary logistic regression was used to identify factors influencing livelihood diversification, while Propensity score matching (PSM) was applied to estimate its causal impact on food security. Robust standard errors were reported to address potential heteroscedasticity, and diagnostic tests confirmed no major multicollinearity or model misspecification issues.

**Key Results:** Among the sampled households, 108 engaged in livelihood diversification. Regression results revealed that education (+15.4%) and household size (+5.9%) significantly increased the likelihood of diversification, whereas access to irrigation (−35.7%), livestock ownership (−2.9%), and credit access (−12.08%) negatively affected it. PSM analysis confirmed a positive and statistically significant impact of diversification on food security, increasing daily kilocalorie intake by 118–136 kcal.

**Conclusion/Policy Implications:** The findings suggest that livelihood diversification significantly enhances food security among smallholder farmers. Therefore, policies should promote diversification through expanded irrigation infrastructure, vocational training (TVET and universities), and support for activities such as animal fattening, dairy farming, and beekeeping. Extension services and microfinance institutions should be mobilized to provide technical and financial support focused on diversified farming strategies.

## 1. Introduction

### 1.1. Background of the Study

Food security refers to the physical and economic access of all people, at all times, to sufficient, safe, nutritious, and healthy food that meets their dietary needs and food preferences for an active and healthy life [1]. In contrast, food insecurity is the inability to obtain adequate food due to financial constraints [2].

Agriculture remains the primary source of food for people worldwide. Both urban and rural populations rely on this sector to meet their nutritional needs. While the majority of agricultural production occurs in rural areas, urban agriculture also plays a significant role. In addition to food production, agriculture contributes to job creation, attracts investment, and generates foreign exchange.

A significant portion of the global population depends on agriculture for their livelihoods, either directly or indirectly. In 2023, it was estimated that between 713 and 757 million people, representing approximately 8.9%–9.4% of the world's population, faced food insecurity. Africa has the highest proportion of people experiencing hunger, at 20.4%, compared to 8.1% in Asia and 7.3% in Oceania. Projections suggest that by 2030, around 582 million individuals will suffer from chronic undernourishment. Additionally, it is estimated that by 2030, 53% of the global population will reside in Africa [3].

The agriculture sector in Africa employs about 42% of the workforce [4], with smallholder farmers contributing approximately 80% of the continent's agricultural output [5]. In developing countries, particularly in sub-Saharan Africa, agriculture is central to rural livelihoods serving as the main source of income, food, employment, and foreign exchange [6]. However, agricultural production in the region is highly dependent on favorable climate conditions and small-scale landholdings [7]. Persistent challenges such as recurrent droughts, ongoing conflicts, low land productivity, and limited access to chemical fertilizers continue to hinder production and exacerbate food insecurity.

In Ethiopia too, agriculture is regarded as the primary pathway out of poverty and food insecurity, having provided the majority of employment and income for the large rural population over the years. Approximately 80% of the population depends on rain-fed agriculture for their livelihoods [8]. In 2020, only about 20% of the total arable land, roughly 11.7 million hectares was cultivated, and nearly 55% of smallholder farmers operated on 1 ha or less [9]. However, the agricultural sector has faced criticism for failing to ensure food security for those who rely on it [10, 11]. Depending solely on agriculture for livelihood has proven inadequate due to shrinking land sizes, fragmented plots, declining soil fertility, low productivity, limited access to organic and chemical fertilizers, and the widespread prevalence of subsistence farming [12, 13]. As a result, rural households are increasingly compelled to diversify their livelihood strategies in order to achieve food security [10, 14].

Despite the government's implementation of various policies such as market liberalization, structural adjustment programs, and the Agricultural Development Led Industrialization (ADLI) Plans I and II aimed at boosting agricultural productivity between 1991 and 2016, Ethiopia's agricultural production remained low and predominantly focused on on-farm development [15, 16]. These policies have largely prioritized on-farm activities, while overlooking the importance of diversifying into off-farm and nonfarm economic opportunities [15, 17]. Sole reliance on on-farm agricultural practices has proven insufficient for achieving food security, especially as population growth outpaces the land's carrying capacity, land productivity remains low, and inflation continues to rise [18]. However, there is a significant research gap in understanding the role and potential of livelihood diversification beyond on-farm activities among smallholder farmers in addressing these persistent challenges.

Currently, in the Amhara region, ongoing conflict between the government and Fano militia, fertilizer shortages, low land productivity, and the presence of uncultivated land due to civil unrest are threatening the food security of the population, particularly rural smallholder farmers. Previously, the COVID-19 pandemic also contributed to food insecurity in the region [8]. To cope with food insecurity, rural smallholder farmers have adopted livelihood diversification strategies [10, 19]. Livelihood diversification refers to the pursuit of a varied portfolio of economic activities and social support mechanisms intended to improve livelihoods and enhance resilience during droughts and other shocks [20]. Abebe et al. [10] and Yenesew and Masresha [14] highlighted that off-farm economic activities significantly reduce food insecurity and poverty. Similarly, Abera et al. [12] reported that on-farm, off-farm, and nonfarm economic activities positively affect the food security status of smallholder farmers in the Chewaka resettler communities of southwestern Ethiopia. However, these studies were conducted in areas distant from the current study location, leaving a research gap in West Gojjam Zone.

Although there is growing evidence of rural households' participation in livelihood diversification, the determinants of such participation vary across regions, depending on local context and asset endowments [12, 15]. Generally, rural farmers diversify their livelihoods through on-farm, off-farm, and nonfarm economic activities. On-farm activities include both crop production and livestock rearing. Off-farm economic activities refer to agricultural work conducted outside the household's farm such as wage labor, land rental, and environmental gathering (e.g., firewood collection and charcoal selling). Nonfarm activities, on the other hand, are nonagricultural and include petty trade, rural crafts, and remittances [14, 15]. Rural households pursue livelihood diversification to manage risk, purchase farm inputs more confidently, reduce income variability, ensure household survival, and increase overall income [21, 22].

There are conflicting findings in previous studies. Some studies stated the evidence of livelihood diversification among smallholder farmers [12, 14, 15, 21]. In particular, Alamneh et al. [15] acknowledged the presence of livelihood diversification in periurban areas of the West Gojjam Zone. Other studies focused on the potential of the West Gojjam Zone for livelihood diversification such as the existence of irrigation [23]. By contrast, other studies pointed to the predominance of traditional rain-fed agriculture only [8, 10, 13]. Some other studies concluded that livelihood diversification is area- and context-specific, suggesting the necessity for area-specific studies to identify the factors determining livelihood diversification in different spatial dimensions [14, 24–26]. In the selected areas of Jabithenan and Bure, no previous studies have focused on livelihood diversification and its impact on food security. Hence, there is a need to investigate the determinants of livelihood diversification in West Gojjam Zone which may not have adequately captured in previous studies regarding the contextual factors affecting food security.

There are studies that revealed the existence of food insecurity in the West Gojjam Zone [10, 23]. Previous studies that identified the presence of livelihood diversification in West Gojjam Zone did not investigate its effect on food security [12, 14, 15, 21]. These previous studies did not study the contribution of livelihood diversification to the food security of smallholder farmers. Hence, there are insufficient studies investigating the determinants and impacts of livelihood diversification on the food security of smallholder farmers in the West Gojjam Zone [27]. Therefore, it is crucial to analyze both the determinants of livelihood diversification and its effects on food security within the local context of West Gojjam Zone.

Accordingly, this study is aimed at the following: (1) assess the determinants of livelihood diversification strategies, (2) examine the impact of livelihood diversification on the food security of rural households, (3) explore strategies to enhance food security, and (4) address the existing information gap on livelihood diversification and food security in the West Gojjam Zone.

## 2. Materials and Methods

### 2.1. Description of the Study Area

**Figure 1:**
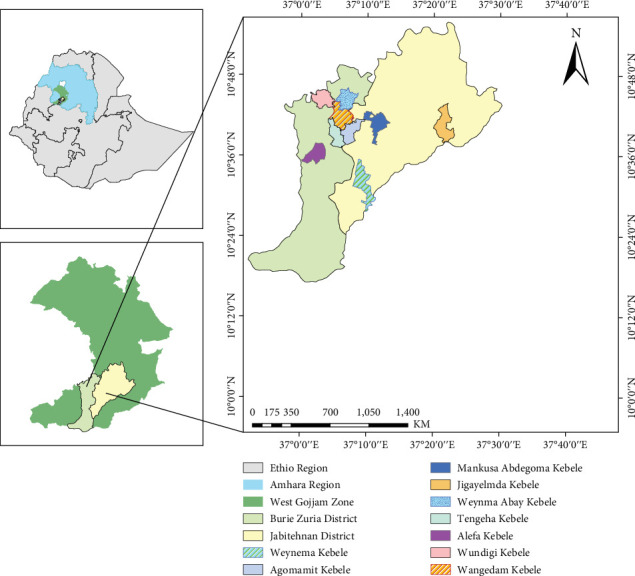
Map of the study area. *Source:* own construction by ArcGIS (2024).

West Gojjam is one of the zones in the Amhara Region, Ethiopia, which is located approximately 350 km northwest of Addis Ababa and to the south of Bahir Dar. West Gojjam is bordered by the Abay River on the south, which separates it from the Oromia region and Benishangul gumuze region; on the northwest by Alefa; on east by East Gojjam; on the north by North Gojjam; and on the west by the Awie zone. In the study area, there are 14 rural Woreda administrations and 6 city administrations [23]. The study area map is presented in Figure 1.

The altitude of the zone ranges from 750 to 3535 m above sea level and receives a mean annual rainfall of 1500 mm, which occurs mainly in June, July, August, and September. The other months of the year are nearly dry with erratic rainfall. The average daily temperature ranges from 14°C to 37°C. The agro-ecological conditions of the study area include 72% Woyina Dega (midhighlands), 16.6% Dega (highlands), 11.2% Kola^1^, and 0.2% Wurch^2^ [28] as cited in Worku [23]. According to the 2015 national census conducted by the CSA of Ethiopia, west Gojjam had a total population of 2,106,596, of whom 1,058,272 are men and 1,048,324 are women. With an area of 13,311.94 km^2^, West Gojjam has a population density of 158.25/km^2^, 184,703 or 8.77% of whom are urban inhabitants.

The most commonly raised livestock include cattle, sheep, goats, donkeys, horses, honeybees, and chickens. Cattle and sheep graze freely on available pasture and produce complementary crops and residues during the dry season, while chickens scavenge and feed on leftover grains. Livestock play a crucial role in household livelihoods, primarily by generating income through the sale of sheep and goats.

In the West Gojjam Zone, households engage in various forms of livelihood diversification, including crop diversification, livestock diversification, and seasonal employment in towns as daily laborers. Agriculture, encompassing both crop production and livestock rearing, is the primary economic activity in Burie Zuria District and Jabithenan District. The region cultivates a variety of crops, particularly commercial ones like wheat (for macaroni, pasta, and bread), teff, beans, lentils, maize, barley, and sorghum, which largely depend on seasonal conditions. However, due to the limited number of irrigation schemes, the production of vegetables and fruits is constrained [15, 23].

### 2.2. Research Design

A cross-sectional research design was used for this study. The study was conducted at the household level. The head of the household was asked about the determinants of livelihood diversification and its impact on food security via questionnaires prepared by the researcher. Both quantitative and qualitative methods were also applied in this study. Quantitative data is generated by questionnaires from respondents for use in inferential statistics to infer sample characteristics of the population. Qualitative data were also employed to assess the subjective opinions, attitudes, and perceptions of the sample respondents. A questionnaire and interviews were also employed to collect data for the study.

### 2.3. Data Type and Source

Primary and secondary data were used to achieve the objective of the study. Primary data were collected from households via structured questionnaires and interviews were used. The interviews were employed to reduce the nonresponse rate. Published and unpublished materials, including records from the district agricultural bureau and data on routinely consumed food items, as well as information from websites, were used as sources of secondary data in this study.

### 2.4. Sampling Technique and Sample Size

A multistage sampling technique was employed to select representative sample households. The Amhara region was chosen due to its status as one of the most food-insecure regions in Ethiopia, second only to the Tigray region, and exacerbated by ongoing conflict and civil war. Within this region, the West Gojjam Zone was selected based on evidence of food insecurity [29]. This zone faces challenges related to agricultural production, climate variability, and other socioeconomic factors that contribute to food insecurity. Among the districts in the West Gojjam Zone, Burie Zuria and Jabithenan districts were selected due to their potential for smallholder farmers to engage in diverse livelihood activities and the presence of food-insecure households [15, 23]. The zone has relatively better infrastructure for irrigation, large grazing lands for dairy and animal fattening, and several Technical and Vocational Education and Training (TVET) colleges and universities that contribute to knowledge diffusion, yet it continues to experience significant food insecurity. This situation makes the study area selection more relevant to the purpose of the study. In the fourth stage, five kebeles^3^ were randomly selected from the Burie Zuria district and four from the Jabithenan district. Finally, representative households were randomly selected from each kebele, ensuring a diverse and representative sample.

From 9 kebeles in both districts, 390 representative sample households were selected based on their proportions in their population as shown in Table 1. The total required sample size was determined via the Yamane formula;

 $$\begin{array}{c} n=\frac{N}{1+N\left(e^{2}\right)}n=\frac{17,500}{1+17,500{\left(0.05\right)}^{2}}=390, \end{array}$$

where *N* = 17,500 is the total number of households, *n* is the sample size = 391, and *e* is the margin of error = 5%.

## 3. Data Analysis

### 3.1. Method of Data Analysis

The study employed both descriptive and econometric methods of data analysis. The descriptive method of data analysis used the mean, standard deviation, minimum, and maximum for a better understanding of the socioeconomic and demographic characteristics of the rural households in the study.

### 3.2. Measuring Livelihood Diversification

Livelihood is defined in many ways. Livelihood is more than just income [30]; livelihood includes income (in cash and kind), social institutions (such as kin, family, compound, and village), property rights, and gender relations [20]. Livelihood also means obtaining access to and benefitting from social and public institutions (such as education, health services, roads, and water supplies) [31]. Diversification, on the other hand, is a strategy used by rural households to increase income and thereby reduce environmental risk [32]. Ellis [20] defined livelihood diversification as the process by which rural households design a diversified portfolio of assets and social capabilities to improve their standard of living.

However, in this study, livelihood diversification is the combination of on-farm, off-farm, and nonfarm activities performed by rural households to obtain income and improve their quality of life. Rural households may generate income from one or a combination of on-farm, off-farm, and nonfarm activities in the study area [15, 32]. The dominant on-farm activities practiced in the study area are crop production and animal rearing. Alamneh et al. [15] noted that the main crops grown in the study area include maize, wheat, sorghum, rice, sesame and soybeans. Moreover, cattle, sheep, goats, donkeys, horses, mules and poultry are major livestock reared by the community. In the study area, nonfarm economic activity practices include rural crafts, petty trade, and remittances from urban areas to rural areas. Off-farm activities are performed outside of households' farm area, such as becoming wage laborers, producing charcoal and selling, gathering and selling firewood and renting land.

In this study, a household is considered diversified in its livelihood if the income obtained from off-farm and nonfarm sources is greater than 50% of the income earned in the year 2022–2023. In contrast, a household is deemed undiversified if the income generated from on-farm economic activities is greater than 50% of the total income received in that same year. The heads of the farm households were asked, through a structured questionnaire, about the estimated income they received in the year 2022/23 from on-farm, off-farm, and nonfarm economic activities. To determine whether a household is diversified or not, this study compared the income from on-farm activities with the income from both off-farm and nonfarm activities ([15, 20, 32, 33].

### 3.3. Measuring Food Security

This study uses the calorie intake method^4^ of calculating physical food consumption as a proxy to calculate food security by households. Physical food consumption statistics are used to assess food security status [8]. To collect data about the calorie intake of physical food consumed by rural households, a structured questionnaire was presented to recall the amount of food consumed and purchased by the family in the past 7 days before the questionnaire was administered. The following steps were performed to transform the data collected into calories for age and gender composition. First, the different local measurements were converted into standard measurements (in terms of grams). Second, the food purchased was converted into the calorie dietary composition table of the Ethiopian Institute of Health and Nutrition Research. Third, the calories in all the food consumed by rural households were added and converted into daily amounts [34]. The aggregate food calories were subsequently adjusted to equivalent forms to those of adults (Adult-equivalent adjustment)^5^ per home [35]. To obtain the daily per capita household calorie intake, the per capita household calorie consumption was divided by 7 days. Fourth, identifying whether a household is food secure or food insecure involves comparing per capita per day of calorie consumption with the daily need of 2200 Kcal per capita per day of calorie consumption defined by the World Health Organisation [8, 10]. Based on Woleba et al. [8] and Abebe et al. [10], households whose daily food intake exceeds the estimated 2200 Kcal per person are classified as food secure or otherwise food insecure.

### 3.4. Econometric Model Specification

Two types of econometric regression models were used in this study. A binary logistic regression models was used to identify the determinants of livelihood diversification, and propensity score matching (PSM)^6^ was used to assess the impact of livelihood diversification on food security.

Livelihood diversification is a binary variable taking a value of 1 if the household is diversified and 0 otherwise.

In accordance with Gujarati and Porter [36], the logistic regression model can be written in terms of the odds ratio and the log of the odds ratio, which helps understand the interpretation of the coefficients. In this study, the odds ratio is the ratio of the probability that the household is diversified (Pi) to the probability that the household is not diversified in terms of livelihood (1-Pi).

 $$\begin{array}{c} \text{Pi}=\left(\text{fi}\right)=f\left(a+\beta \text{iXi}\right)=\frac{1}{1+e^{-\left(a+\beta \text{iXi}\right)}}=\frac{1}{1+e^{-\text{Zi}}}. \end{array}$$

Since Zi = *a* + *β*iXi, the above formula can be written for easy understanding as:

 $$\begin{array}{c} 1-\text{Pi}=1-\frac{1}{1+e^{-\text{Zi}}}. \end{array}$$

The odds ratio then becomes

 $$\begin{array}{c} \frac{\text{Pi}}{1-\text{Pi}}=\frac{1/1+e^{-\text{Zi}}}{1/1+e^{-\text{Zi}}}=e^{\text{Zi}}. \end{array}$$

Therefore, Pi/1 − Pi = *e*^Zi^.

Taking the natural logarithm of equation, Yi = ln(Pi/1 − Pi) = ln(*e*^Zi^) = *e*^Zi^ = *a* + ∑_*i*=1_^*K*^*β*iXi + *μ*i, where *k* is the number of explanatory variables, Xi is the vector of independent variables, *μ*i is the error term, *a* is the value of the log odds ratio when the independent variable is zero, and *β* measures the logit change for a unit change in the explanatory variable.

#### 3.4.1. PSM Technique

The main objective of this study is to examine whether livelihood diversification improves the food security status of rural households. A direct comparison of the difference in food security between diversified households and nondiversified households in terms of livelihood is wrong because the difference may not be exclusively obtained from the use of a livelihood diversification strategy but also from other characteristics of rural households. To check the impact of livelihood diversification on the food security of rural households, PSM was applied. The first task of impact evaluation is to overcome the self-selection problem. PSM solves the self-selection problem by estimating the propensity scores (probabilities of households participating in the livelihood diversification strategy) and matching (finding diversified rural households and nondiversified rural households with equal/similar propensity scores) the propensity scores of the treated and untreated groups of rural households within the common support region [37]. The treated group of rural households has diverse livelihoods, whereas the control groups include rural households whose livelihoods are not diverse. The steps used in PSM were estimation of the propensity scores, choosing the matching algorithm, checking the common support condition, and testing the matching quality.

The propensity score can be written as follows: *P*(*x*) = *P*(*T* = 1/*X*) = *E*(*T*/*X*); *P*(*X*) = *Z*(*H*(Xi)), where *T* is an indicator of treatment (diversified households), *X* is a vector of the observed variables, and *Z*(.) is either a logistic or a normal distribution. For simplicity, the propensity score was estimated via logit regression.

The average treatment effect on the treated (ATT)^7^ was calculated on the basis of the propensity scores. The ATT group is the mean outcome in food security difference between those who were diversified in their livelihood and those who were not diversified with similar propensity scores. Mathematically written as

$$\begin{array}{cc} \; & \text{ATT}=E\left\{\frac{\text{Yiu}-\text{Yin}}{T=1}\right\},\\ \text{ATT}=E\left\lceil E\left\{\frac{\text{Yiu}-\text{Yin}}{T=1,\text{P}\left(\text{X}\right)}\right\}\right\rceil ,\\ \text{ATT}=E\left[E\left\{\frac{\text{Yiu}}{T=1,\text{P}\left(\text{X}\right)}\right\}-E\left\{\left(\frac{\text{Yin}}{T=0,P\left(X\right)}\right.\right\}\right], \end{array}$$

where Yiu and Yin are the mean outcome values for diversified and nondiversified rural households, respectively. *E*{Yiu/*T* = 1, *P*(*X*)} represents the mean outcome of diversified rural households, and *E*{(Yin/*T* = 0, *P*(*X*)} represents the mean outcome of nondiversified households (counterfactual situation).

The average treatment effect of livelihood diversification (ATT) estimates the difference only in the common support region^8^, which contains the minimum and maximum propensity scores of the treatment and control groups of rural households, respectively.

The next step was to choose among the alternative matching algorithms^9^. There are alternative matching algorithms, such as nearest neighbor matching (NNM), kernel-based matching, and caliper matching. There is no single recommended matching algorithm, and each algorithm has its advantages. In this study, nearest-neighbor matching and kernel-based matching with different bandwidths were applied.

Finally, to test the matching quality, the lower the pseudo-*R*^2^ is, the insignificant the likelihood ratio is, the greater the reduction in the mean standardized bias^10^ after matching is, and the greater the likelihood ratio results in a large matched sample size, which indicates the quality of the matching procedure applied in this study [37].

### 3.5. Definitions of Variables and Measurements

The dependent variable was livelihood diversification. Livelihood diversification was measured as a dummy variable taking a value of 1 for diversified households and 0 for undiversified households.

The outcome variable was food security. Food security was measured by household caloric intake per day per adult equivalent. A person taking 2200 Kcal and above per day was considered food secure; otherwise, a person was considered food insecure.

Explanatory variables are listed in Table 2.

## 4. Results and Discussion

### 4.1. Livelihood Diversification Status of Households With Continuous Variables

Out of the total sampled households, 27.7% were identified as diversified in their livelihood which means they derived more than half of their income from off-farm and nonfarm sources, while the remaining 72.3% depended primarily on on-farm income. This indicates that a large majority of households in the study area remain reliant on traditional on-farm agriculture activity, indicating a limited shift toward diversified livelihood strategies (Table 3). This indicates that to make livelihood diversification adopted by the majority of smallholder farmers, a stronger diversification promotion strategy is needed.

In terms of food security, measured by daily kilocalorie intake, there was no statistically significant difference between diversified and nondiversified households. This suggests that livelihood diversification alone may not automatically translate into food security unless the new income source from diversification is stable, sufficient, and reliable. The mere existence of diversification is not a guarantee for food security but its sustainability and quality matter. This finding contrasts with studies such as Gebre et al. [41], which found a positive link between income diversification and improved food security, indicating that local context and the quality of diversification matter significantly.

Age and household size did not differ significantly between the two groups. This implies that demographic characteristics may not be the determining factor of diversification in the study area. This implies that policies aimed at promoting diversification based solely on age or household size may be ineffective. These findings contrast with those of Abebe et al. [10], who found that younger and larger households were more likely to diversify. In the context of this study, however, other factors particularly livestock ownership and possibly access to economic opportunities appear to play a more critical role in influencing diversification behavior.

Landholding size was slightly higher for diversified households, although the difference was not statistically significant. This suggests that land size alone may not be a decisive factor in diversification. However, the study by Gebre et al. [41] notes that land fragmentation and tenure insecurity can limit the potential for diversification, focusing on the need for land policy reforms to support diversified livelihoods.

Livestock ownership showed a statistically significant difference between diversified and nondiversified households. Households with more livestock were more likely to diversify their livelihoods. This suggests that livestock can act as a key asset that either enables investment in alternative income activities or provides insurance that allows households to take on new economic risks. This finding supports arguments made by Gebre et al. [41], who identified asset ownership as a key enabler of livelihood diversification in rural Ethiopia.

Distance to the nearest market was not a significant differentiating factor between diversified and nondiversified groups. Both diversified and nondiversified households were, on average, located around 4.3–4.4 km from the market. Indicating that physical access to markets alone may not be a sufficient condition for diversification. This suggests that physical proximity to markets alone does not guarantee participation in off-farm or nonfarm activities. This may reflect other barriers such as market information; lack of skills, capital, or social networks must be combined with physical market proximity to enable smallholder farmers to participate in diversification. This aligns with the findings by Taye et al. [33], who noted that nonspatial barriers often restrict rural households from engaging in diversified livelihood options.

### 4.2. Livelihood Diversification Status and Dummy Variable Characteristics

First, food security status does not appear to be strongly associated with livelihood diversification in this sample. Both diversified and nondiversified households are found in the food secure and insecure categories, suggesting that diversification alone does not guarantee improved food security. This finding aligns with Gebre et al. [41], who note that while diversification often contributes positively to food security, the extent of its benefit depends on the type and profitability of the alternative income sources pursued (see Table 4).

Regarding gender, the majority of households were male-headed, yet the diversification patterns between male and female heads did not differ significantly. This reflects structural gender inequalities in access to resources and economic opportunities that persist across both groups [10]. The lack of statistical significance suggests that both men and women face similar barriers or enabling conditions for diversification in the study area.

Similarly, the level of education did not exhibit a statistically significant association with diversification. While it is conventionally assumed that education facilitates diversification into off-farm and nonfarm activities, the current findings suggest the absence of rural labor markets, limited private sector presence, and weak linkages between education and employment that reduce the returns on education for diversification efforts [12, 39].

In contrast, access to irrigation was significantly associated with diversification. Households with irrigation access were much more likely to diversify their livelihoods. This supports the view that access to productive infrastructure can enable households to engage in higher-value agricultural or allied activities, thereby reducing their dependence on rain-fed farming. Irrigation enables smallholder farmers to practice mixed farming. This signals the need to invest in small-scale irrigation and water management infrastructure to support sustainable diversification [32, 39].

Moreover, credit access was another variable significantly associated with livelihood diversification. Households with credit were more engaged in off-farm and nonfarm activities. This aligns with previous studies indicating that access to financial capital enables smallholder farmers to purchase inputs and engage in nonfarm and off-farm activities without liquidity constraints [8, 25]. These findings highlight the need to expand access to microfinance products by promoting inclusive financial systems that specifically target the rural poor, particularly through the provision of low-interest credit and minimal collateral requirements [42].

### 4.3. Determinants of Livelihood Diversification

Table 5 shows the results of the determinants of livelihood diversification as estimated via binary logit regression. The educational level of the household head, household size, irrigation access, livestock ownership and credit access were statistically significant at the 1% level of significance, whereas the age of the household head, sex of the household head, landholding size and market distance was statistically insignificant at influencing livelihood diversification in the study area.

The marginal effect results indicate that the educational level of the household head increases livelihood diversification by farm households. The results show that the probability of livelihood diversification by literate household heads was 15.4% greater than that of illiterate heads. Literate households use their knowledge, experience and strong linkages with people to develop diverse livelihood strategies. Literate households are more likely to search for information about profitable livelihood opportunities than illiterate ones, which increases their rate of adoption of livelihood diversification strategies. This result is consistent with previous findings [10, 12]. This signals the importance of providing short-term training such as workshops, field visits, and demonstrations to raise awareness and build the capacity of smallholder farmers to develop diverse livelihood strategies.

Household size positively influences the livelihood diversification strategy of households in the study area at the 1% level of significance. As the household size increases by one member, the probability of livelihood diversification increases by 5.9%. In the rural area of the study area, every household member except the very baby had been engaged in income-generating activities. That is, as household size increases, the labor force that participates in different activities increases, which means that households diversify their livelihoods. This result is consistent with the findings of [12]. In addition, if vocational training is provided to youth and adults, households with larger family sizes can benefit through sustainable income growth by diversifying their livelihoods into areas such as handicrafts and weaving, which are compatible with on-farm activities.

Access to irrigation was another variable that influenced livelihood diversification in the study area. The results indicated that households with access to irrigation for their agricultural activities decrease the probability of livelihood diversification by 35.7% than households with no access to irrigation. This may be because households that use irrigation are more likely to engage in agricultural (on-farm) activities than in off-farm and nonfarm activities. To use the irrigational water farmers spent their time and focused on producing twice if possible three times a year rather than shifting to off-farm and nonfarm activities. On the other hand, households with no access to irrigation find themselves participating in off-farm and nonfarm activities to generate more income and thereby diversify their livelihood in seasons other than during the summer rainy season. This result is congruent with previous results [14, 27, 43]. However, households with access to irrigation can manage their on-farm activities alongside nonfarm and off-farm engagements, even though multiple harvests per year may itself be considered a form of livelihood diversification. This highlights the need to expand irrigation infrastructure in food-insecure areas through collaborative efforts by the government, NGOs, and private entrepreneurs.

Livestock ownership by households negatively influences livelihood diversification at the 1% level of significance. When livestock ownership increases by one more livestock, the probability of livelihood diversification by households decreases by 2.9%. Households who own more livestock spend more time rearing livestock and have no extra time engaging in nonfarm and off-farm activities. That is, households with more livestock tend to engage in diversified livestock-based activities such as animal fattening, dairy farming, and beekeeping which enhance the income sources of smallholder farmers. Although this contributes to income generation, such internal (sectoral) diversification appears to reduce the likelihood of engaging in off-farm and nonfarm activities. This may be due to labor or time constraints, or a perceived comparative advantage in livestock activities. Despite this, the result points to the need to focus on a balance between livestock diversification and other broader livelihood strategies such as on-farm and nonfarm diversification. The result is as expected and is consistent with the result of [27, 40]. But in contrast to the findings of Abebe et al., [10] and Gebre et al., [41].

Finally, access to credit negatively influences livelihood diversification by households at the 1% level of significance. The results revealed that access to credit decreases the probability of livelihood diversification by 12% compared to households without access to credit. This unexpected finding may be attributed to the tendency of credit-accessing households to use borrowed funds primarily for purchasing agricultural inputs, thereby focusing more on on-farm activities rather than diversifying into off-farm or nonfarm sectors. In addition, households in the study area face significant barriers to credit access, including high interest rates and the requirement of high-value collateral. Consequently, many are discouraged from borrowing, and those who do often resort to using new loans to repay previous debts instead of investing in livelihood diversification. This outcome contrasts with prior expectations [44]. The findings of this study imply the need to reform credit management systems. Specifically, creditors should closely monitor how loans are utilized by smallholder farmers to ensure that funds are directed toward productive and income-generating activities, thereby reducing debt dependency and encouraging diversification.

Model diagnostic tests were conducted. The econometric model results were found free from multicollinearity and model misspecification and the results were reported using robust standard errors to correct for possible heteroscedasticity.

### 4.4. Impact of Livelihood Diversification on Food Security

The PSM model is the second type of regression model used in this research to investigate the impact of livelihood diversification on the food security of rural households.

The next step is to match farm households of diversified households (treated group) with nondiversified households (control group) with different socioeconomic characteristics based on propensity scores.

#### 4.4.1. Common Support

**Figure 2:**
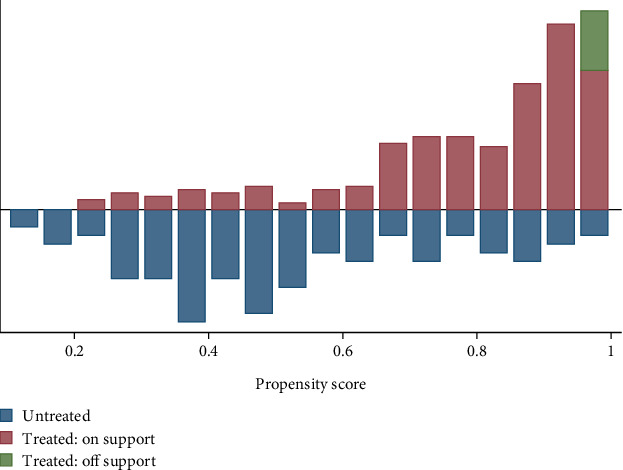
Graphical expression of common support. *Source:* own manipulation of data by Stata (2024).

The common support variable reflects the probability that households will diversify their livelihoods. The common support figure is shown in Figure 2 below. The figure shows that the common support region lies between 0.1297252 and 0.9977594, where 372 (95.38%) out of 390 observations were on the common support and only the remaining 18 (4.62%) were off the common support. Therefore, based on the common support criteria shown in Figure 2, it is possible to perform PSM estimation.

The remaining 2 out of 390 observations or 4.62% exist either below 0.1297252 or above 0.9977594. As indicated in Figure 1, the common support condition was satisfied since there was considerable overlap in the distribution of the propensity scores of both those who were diversified in the upper part of the graph and nondiversified rural households in the lower part of the graph.

#### 4.4.2. Matching Algorithm Selection

Before the estimation of the impact of livelihood diversification on household food security, the quality of alternative matching algorithms was checked based on mean standardized bias, pseudo *R*^2^ and likelihood ratio tests before and after matching. As shown in Table 6, the mean standardized bias was 24.7% before matching and was reduced to 18%–12.2%, with a substantial reduction in standardized bias ranging from 27.12% to 50.61%. The pseudo-*R*^2^ was 0.235% before matching and was reduced to 0.035%. Moreover, the likelihood ratio tests revealed the joint insignificance of the covariates after matching, whereas it was significant before matching. Hence, low mean standardized bias, high total reduction of bias, low pseudo R^2^, and insignificant *p* values of the likelihood ratio test after matching suggest that the PSM procedure is reasonably successful.

After confirming the quality of the matching, the ATT was estimated using both nearest neighbor and kernel matching algorithms. The ATT, which measures the effect of livelihood diversification on the food security of rural households in the study area, is estimated via NNM and kernel matching algorithms. Similar results generated by the different algorithms indicate the robustness of the results. As indicated by Table 7, the food security of households with livelihood diversification increased by 136 Kcal when NNM-1 was used at a significance level of 1%. That is, when NNM-1 was used, households whose livelihoods did not diversify could have increased their food intake by 136 Kcal if they had diversified their livelihoods. NNM-5 revealed an increase in 118-food kilocalorie intake at the 1% level of significance. This also means that households with no livelihood diversification could have increased food kilocalorie intake by 118 as per the NNM-5 estimator. The KBB reported an increase of 136 in the food kilocalorie intake of those who had diversified their livelihood. The difference in income was significant at the 1% level. The results of all four algorithms reveal significant differences in the levels of food security among households. The difference is therefore attributable to the treatment variable, which is livelihood diversification. The results therefore indicate that livelihood diversification has a significant effect on increasing the food security of households. This aligns with the theory that income smoothing through livelihood diversification contributes to improved food security outcomes in vulnerable spatial areas.

This finding is in line with previous studies, such as Kassegn and Endris [45], who found that diversified income sources enhance household food access in Ethiopia's drought-prone regions. Similarly, Yenesew and Masresha [14, 46] and Zeleke et al. [18] reported that households practicing off-farm and nonfarm activities were less food insecure than those relying solely on agriculture. Furthermore, the findings of this study are supported by Mahedi et al. [26, 44], who observed similar effects in agroecological zones where climatic variability forces households to seek diversified sources of income.

However, while the results of this study align with earlier findings, this study makes a unique contribution by applying a rigorous matching algorithm to control for selection bias in the estimation of food security impacts in the Jabithenan and Bure Zuria district context. The use of multiple matching techniques strengthens the reliability of these conclusions and provides evidence tailored to the West Gojjam Zone rural setting.

These findings suggest that promoting livelihood diversification through skills training, credit access, and market linkages can serve as an effective strategy to enhance food security. Policymakers should therefore prioritize interventions that expand rural households' options beyond traditional agriculture.

## 5. Conclusion and Recommendations

This study examined the impact of livelihood diversification on rural household food security in the West Gojjam Zone and identified key factors influencing diversification. Using a mixed-methods approach and PSM, the study found that educational level, household size, livestock ownership, access to irrigation, and credit significantly influence engagement in diversified livelihoods. The results show that only 27.7% of households were diversified, yet 71.03% were food secure. Diversified households consumed approximately 118–136 Kcal more per day compared to nondiversified ones.

These findings strengthen the growing consensus that livelihood diversification plays a positive role in enhancing rural food security by reducing household vulnerability to climate shocks and market fluctuations. However, a key limitation of this study is its reliance on cross-sectional data, which restricts the ability to capture seasonal variations and long-term dynamics in food security outcomes.

Based on the study's findings, several actionable recommendations are proposed to enhance rural household food security through livelihood diversification. First, expanding access to productive infrastructure, particularly irrigation systems, is crucial for improving food security. Irrigation enables multiple harvests per year and reduces dependence on erratic rainfall. Collaborative efforts among government agencies, nongovernmental organizations (NGOs), and private investors should focus on constructing and upgrading irrigation infrastructure in rural areas. Second, agricultural diversification should be promoted through the integration of crop production with livestock activities such as animal fattening, dairy farming, and beekeeping. These practices not only increase household income but also build resilience against agricultural shocks. Extension services should play a central role by offering technical guidance, regular follow-ups, and tailored demonstrations to smallholder farmers.

Third, rural households should be encouraged and supported to engage in off-farm and nonfarm income-generating activities such as weaving, handicrafts, and small-scale food processing. These activities are compatible with agricultural schedules and can provide alternative income sources, especially during the off-season. To support this, training and skill development programs should be delivered through TVET colleges, universities, and agricultural extension services, with a particular focus on food-insecure regions. Fourth, access to inclusive and affordable financial services should be strengthened. Microfinance institutions, banks, and NGOs should provide rural households with low-interest loans and flexible repayment terms to reduce financial barriers and encourage investment in diverse livelihood activities. These financial services should be tailored to the needs of smallholder farmers and entrepreneurs in food-insecure areas.

Lastly, special attention should be given to empowering women and youth through targeted training and capacity-building programs. Engaging these groups in diversified livelihood activities not only enhances household income but also contributes to social inclusion and sustainable development. Extension workers and development agents should provide continuous education on the importance of diversification and conduct regular field visits to offer feedback and technical support to households engaged in practices such as animal fattening, dairy production, irrigation farming, and handicrafts.

### 5.1. Future Research Directions

Future studies should focus on the longitudinal effects of livelihood diversification on household food security to assess seasonal and long-term impacts. Furthermore, exploring the role of climate-smart agriculture technology practices in enhancing the effectiveness of diversification and their impact on food security could also be helpful for smallholder farmers to achieve sustainable food security.

## Figures and Tables

**Table 1:** The proportional sample size determination of the target groups.

	**Kebele**	**Total household**	**Sample size**	**Remark**
Burie Zuria district	Wangedam	1890	42	$42=\frac{1890*390}{17,500}$
	Wundigi	1682	37	$37=\frac{1682*390}{17,500}$
	Tengeha	2656	59	$59=\frac{2656*390}{17,500}$
	Alefa	1461	33	$33=\frac{1461*390}{17,500}$
	Weynema Abay	1928	43	$43=\frac{1928*390}{17,500}$

Jabitehnan district	Mankusa Abdegoma	1682	38	$38=\frac{1682*390}{17,500}$
	Jigayelmda	2613	58	$58=\frac{2613*390}{17,500}$
	Weyenema	1860	41	$41=\frac{1860*390}{17,500}$
	Agomamit	1728	39	$39=\frac{1728*390}{17,500}$

	Total	17,500	390	

*Note: Source:* district administration (2024).

**Table 2:** Summary of the variables used in the econometric regression.

**Treatment variables**	**Description**	**Measurement/category**	**Applicable references** ^**a**^	**Expected sign**
Age	Age of household head	Continuous (years)	[1], [2], [3]	−
EDU	Education level of household head	Dummy (1 = literate, 0 = illiterate)	[3], [2], [4]	+
Sex	Sex of household head	Dummy (1 = male, 0 = female)	[3], [5], [6]	+
HHS	Household size	Continuous (number of members)	[1], [7], [8]	+
Credit	Access to credit	Dummy (1 = accessed credit, 0 = otherwise)	[9], [10]	+
Irrigation	Access to irrigation	Dummy (1 = uses irrigation, 0 = otherwise)	[11], [2]	+
Livestock (TLU)^b^	Livestock ownership	Continuous (TLU)	[12], [11]	+
LHS	Landholding size	Continuous (hectares)	[11], [3], [12]	+
Market distance	Distance from home to the nearest market	Continuous (kilometers)	[10], [12], [11]	− or +

*Note: Source:* own compilation (2024).

^a^Applicable references: (1) Yussuf and Mohamed [38], (2) Ayana et al. [39], (3) Alamneh et al. [15], (4) Abera et al. [12], (5) Abebe et al. [10], (6) Yenesew and Masresha [14], (7) Worku [23], (8) Demeke [40], (9) Woleba et al. [8], (10) Gebrehiwot et al. [25], (11) Admasu et al. [32], and (12) Taye et al. [33].

^b^Tropical livestock unit (TLU) refers to a standardized measure that converts different types of livestock owned by a farmer into a common unit, allowing for uniform comparison across livestock species.

**Table 3:** Summary statistics of continuous variables with livelihood diversification status.

**Variables**	**Diversification status**		**Combined**	**T** ** test (** **p** ** value)**
	**Diversified (=108)**	**Nondiversified (=282)**		
	**Mean Std.dev**	**Mean Std.dev**	**Mean Std.dev**	
Food security (Kcal intake)	2580.833 (496.5583)	2549.84 (521.3689)	2558.423 (514.1718)	0.5322 (0.2975)
Age	40.7963 (14.77543)	41.29433 (15.98471)	41.15641 (15.642)	0.2810 (0.6106)
Household size	1.555556 (0.7015781)	1.659574 (0.9228595)	1.630769 (0.867628)	1.0596 (0.8550)
Landholding size	2.94213 (1.947202)	2.742021 (1.67451)	2.797436 (1.753987)	1.0082 (0.1570)
Livestock ownership	12.17593 (4.251066)	9.929078 (5.143694)	10.55128 (5.009624)	4.0408 (0.0000)
Market distance	4.361111 (1.66019)	4.283688 (1.847673)	4.305128 (1.795946)	0.3805 (0.3519)

*Note: Source:* own calculation of data (2024).

**Table 4:** Summary statistics of dummy variables with livelihood diversification status.

**Variables**		**Diversification status of household**		**Combined**	**Chi-square (** **p** ** value)**
		**Diversified = (108)** **Mean (std.dev)**	**Nondiversified = (282)** **Mean (std.dev)**		
Food security	Secure	80 (=20.51)	197 (=50.51)	277 (=71.03)	0.6745 (0.411)
	Insecure	28 (=7.18)	85 (=21.79)	113 (=28.97)	

Sex	Male	92 (=23.59)	240 (=61.54)	332 (=85.13)	0.0004 (0.984)
	Female	16 (=4.10)	42 (10.77)	58 (14.87)	

Education	Literate	12 (=3.08)	47 (=12.05)	59 (=15.13)	1.8772 (0.171)
	Illiterate	96 (=24.62)	235 (=60.26)	331 (=84.87)	

Irrigation	Users	10 (=2.56)	123 (=31.54)	133 (=34.10)	41.0206 (0.000)
	Nonusers	98 (=25.13)	159 (=40.77)	257 (=65.90)	

Credit	Access	12 (=3.08)	78 (=20)	90 (=23.08)	12.0473 (=0.001)
	Otherwise	96 (=24.62)	204 (=52.31)	300 (=76.92)	

*Note: Source:* own calculation of data (2024).

**Table 5:** Logit estimate results of the determinants of livelihood diversification.

**Explanatory variable**	**Estimated coefficient**	**Standard error (robust)**	**Marginal effect**	**p** ** value**
Age	−0.006405	0.0084166	−0.0010241	0.447
Sex	−0.1178007	0.3848196	−0.018368	0.754
Education	1.270621	0.4729581	0 0.1540328	0.000⁣^∗∗∗^
Household size	0.3698869	0.1627971	0.0591391	0.018⁣^∗∗^
Land holding size	−0.0685868	0.0712383	−0.010966	0.337
Irrigation	−2.817004	0.4356028	−0.3573348	0.000⁣^∗∗∗^
Livestock ownership	−0.1875532	0.0316708	−0.0299868	0.000⁣^∗∗∗^
Market Distance	−0.046205	0.0759557	−0.0073875	0.543
Credit access	−0.8729227	0.3566065	−0.1208169	0.005⁣^∗∗∗^
Constant	5.853488	0.9025601	—	—
No. of observation 390 Pseudo *R*^2^ 0.2394		Wald chi^2^ (7) (*p* value)	66.21 (0.000)	

*Note: *⁣^∗∗∗^ and ⁣^∗∗^ indicate significance at the 1% and 5% levels of significance, respectively. *Source:* own data regression result by Stata (2024).

**Table 6:** Covariate balance indicators before and after matching.

**Matching algorithm**	**NNM-1**	**NNM-5**	**KBM-0.03**	**KBM-0.06**
Mean standard bias (before)	24.7	24.7	24.7	24.7
Mean standard bias (after)	18.0	12.2	18.0	18.0
Percentage of bias reduction	27.12%	50.61%	50.61%	50.61%
Pseudo *R*^2^ (before)	0.235	0.235	0.235	0.235
Pseudo *R*^2^ (after)	0.074	0.035	0.074	0.074
LR *X*^2^ with *p* value (before)	108.18	108.18	108.18	108.18
*P* > *X*^2^	0.000	0.000	0.000	0.000
LR *X*^2^ with *p* value (after)	53.94	25.26	53.94	53.94
*P* > *X*^2^	0.000	0.003	0.000	0.000

*Note: Source:* own calculation of data by Stata (2024).

Abbreviations: KBM-0.03, kernel-based matching with 0.03 bandwidth; KBM-0.06, kernel-based matching with 0.06 bandwidth; NNM-1, nearest neighbors matching with single neighbors; NNM-5, nearest neighbors matching with five neighbors.

**Table 7:** Estimated effects of livelihood diversification on food security.

**Matching**	**Treated**	**Control**	**Mean outcome**		
			**ATT**	***T* value**	**Standard error**
NNM-1	2546.96591	2410.81439	136.151515	1.55⁣^∗∗∗^	88.0023184
NNM-5	2546.96591	2428.675	118.290909	1.32⁣^∗∗^	89.8255773
KBB-0.03	2546.96591	2410.81439	136.151515	1.55⁣^∗∗∗^	88.0023184
KBB-0.06	2546.96591	2410.81439	136.151515	1.55⁣^∗∗∗^	88.0023184

*Note: *⁣^∗∗∗^ and ⁣^∗∗^ represent significance at the 1% and 5% levels, respectively. *Source:* own estimation calculation by Stata (2023).

## Data Availability

Data will be available based on request.

## Endnotes

^1^Kolla refers to the lowland zone in Ethiopia, typically found below 1500 m above sea level, characterized by hot temperatures (usually above 20°C), low to moderate rainfall, and semiarid conditions, and commonly associated with drought-prone area.

^2^Wurch refers to the highland zone in Ethiopia, located at altitudes above 3200 m above sea level, characterized by cold temperatures, frequent frost, and short growing seasons.

^3^Kebele is the smallest administrative unit lower than district.

^4^A method used to assess food security by calculating the total calories consumed per household per day and comparing this to a minimum threshold (2200 Kcal/person/day).

^5^A process of converting household food consumption to a standardized unit by adjusting for age and sex differences in nutritional requirements.

^6^A quasiexperimental technique used to estimate the causal effect of a treatment by matching treated and untreated units with similar characteristics.

^7^The average impact of the treatment (livelihood diversification) on those households who actually received it.

^8^The range of propensity scores where both treated and untreated households can be compared because they share similar covariate values.

^9^Techniques used in PSM to pair treated and untreated units. Examples include nearest neighbor, kernel-based, and caliper matching.

^10^A measure of the difference in covariate means between treated and control groups, used to assess matching quality in PSM.
